# Familial testicular germ cell tumors in two brothers

**DOI:** 10.1002/iju5.12156

**Published:** 2020-04-12

**Authors:** Senji Hoshi, Vladimir Bilim, Kiyotsugu Hoshi, Isoji Sasagawa, Kotarou Otake, Daigo Chiba, Shinta Suenaga, Masahito Konno, Yuki Katsumata, Kento Morozumi, Jun Takemoto, Kenji Numahata, Keiichi Ito

**Affiliations:** ^1^ Department of Urology Yamagata Tokushukai Hospital Yamagata Japan; ^2^ Department of Urology Kameda Daiichi Hospital Niigata Japan; ^3^ Department of Urology Yamagata Prefectural Central Hospital Yamagata Japan; ^4^ Ito Urology Clinic Yamagata Japan

**Keywords:** familial testicular tumor, genetic predisposition, non‐seminoma germ cell tumor, seminoma, testicular cancer

## Abstract

**Introduction:**

Two percent of testicular germ cell tumors occur in family clusters. Here, we report metachronous testicular germ cell tumors in two brothers.

**Case presentation:**

An elder brother was diagnosed at the age of 30 years old and the pathological diagnosis was mixed testicular germ cell tumor. A tumor in the younger brother was suspected during testicular self‐examination. It was confirmed by ultrasound examination at the age of 30 years old, 3 years and 6 months after the diagnosis of the testicular tumor in elder brother. The pathological diagnosis was pure seminoma. Both brothers had stage 1 testicular germ cell tumors and no recurrence was observed during the follow‐up period of 4 years and 4 months and 10 months, respectively.

**Conclusion:**

Various histological types of tumor can occur in members of one family. Besides genetic predisposition, shared diet, environmental exposure and other factors can contribute to the familial testicular cancer. Testicular self‐examination is recommended for family members of a person with testicular germ cell tumor.

**Abbreviations & Acronyms**
AFP: alpha‐fetoprotein
FRR: frailty relative risk
SNP: single‐nucleotide polymorphism
TGCT: testicular germ cell tumor

**Keynote message**

Testicular cancer is the most common solid tumor in young adult males. Familial germ cell tumors comprise 2% of all testicular cancer cases. Here, we report the testicular cancer in two brothers.

## Introduction

Testicular cancer is the most common solid tumor in young adult males.^1^ TGCT occur in 1 in 500 men and account for 1% of all cancers in men. Two to 5% of patients develop cancer of the contralateral testicle.^2^ Epidemiologic studies have demonstrated an increasing incidence of TGCT since the mid‐20th century.^3^, ^4^, ^5^ Familial germ cell tumors comprise 2–5% of all testicular cancer cases.^6^ They occur earlier than sporadic tumors. Risk for testicular cancer is 6–8 times higher in persons whose fathers had testicular cancer.

During the past 20 years, 64 patients were diagnosed with TGCT at our institutions. Among them, there were 47 patients with seminoma and 17 non‐seminoma tumors. Mean age of seminoma patients was 37.6 years and non‐seminoma patients was 31.7 years. Among the patients there were two brothers, diagnosed and treated for TGCT, comprising 3.125% of all testicular tumors.

## Case presentation

**Fig. 1:**
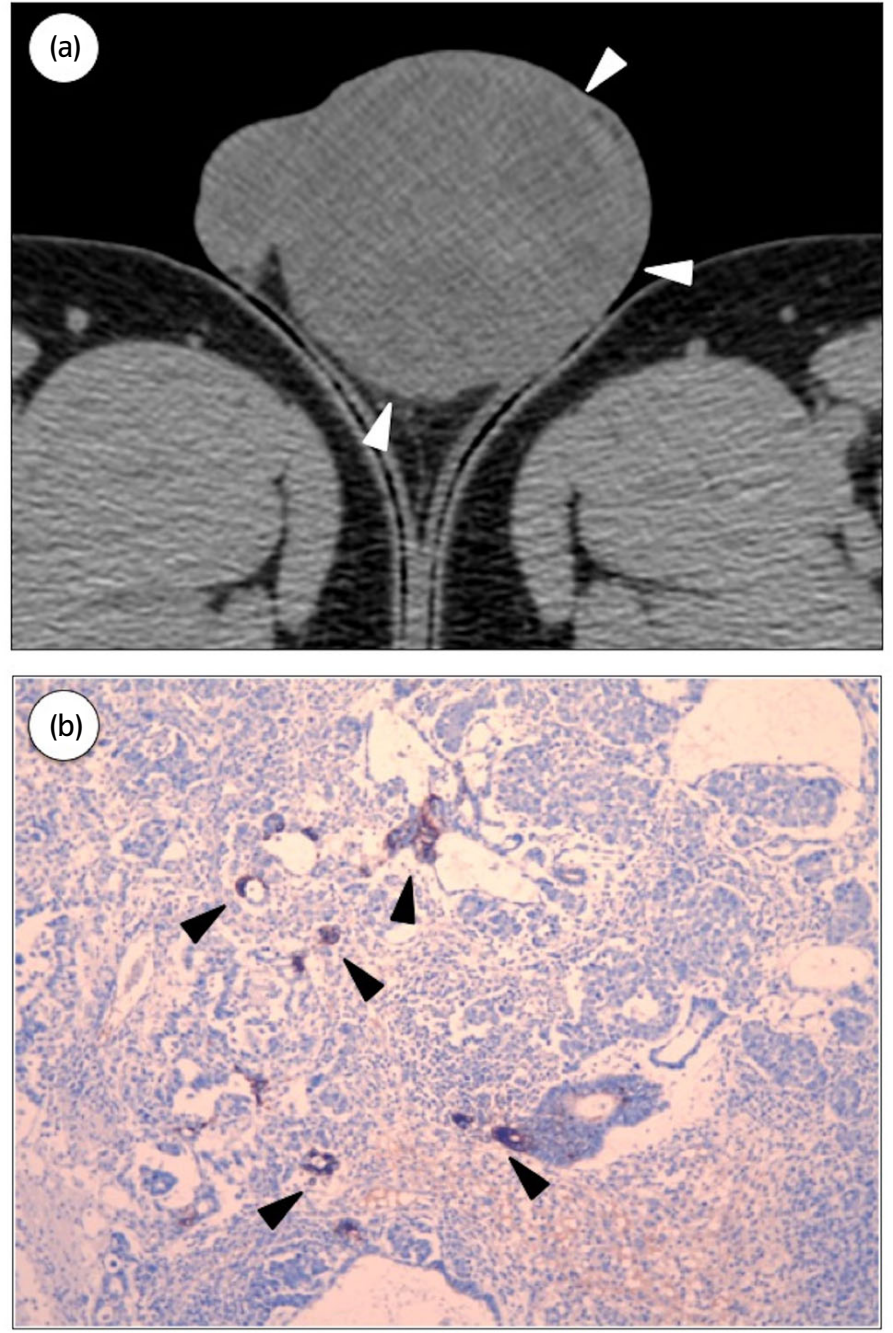
Case 1. (a) Computed tomography of the testes. (b) Immunohistochemical staining for AFP. Mixed germ cell tumor (seminoma, yolk sac tumor). Arrowheads indicate cells positive for AFP.

**Fig. 2:**
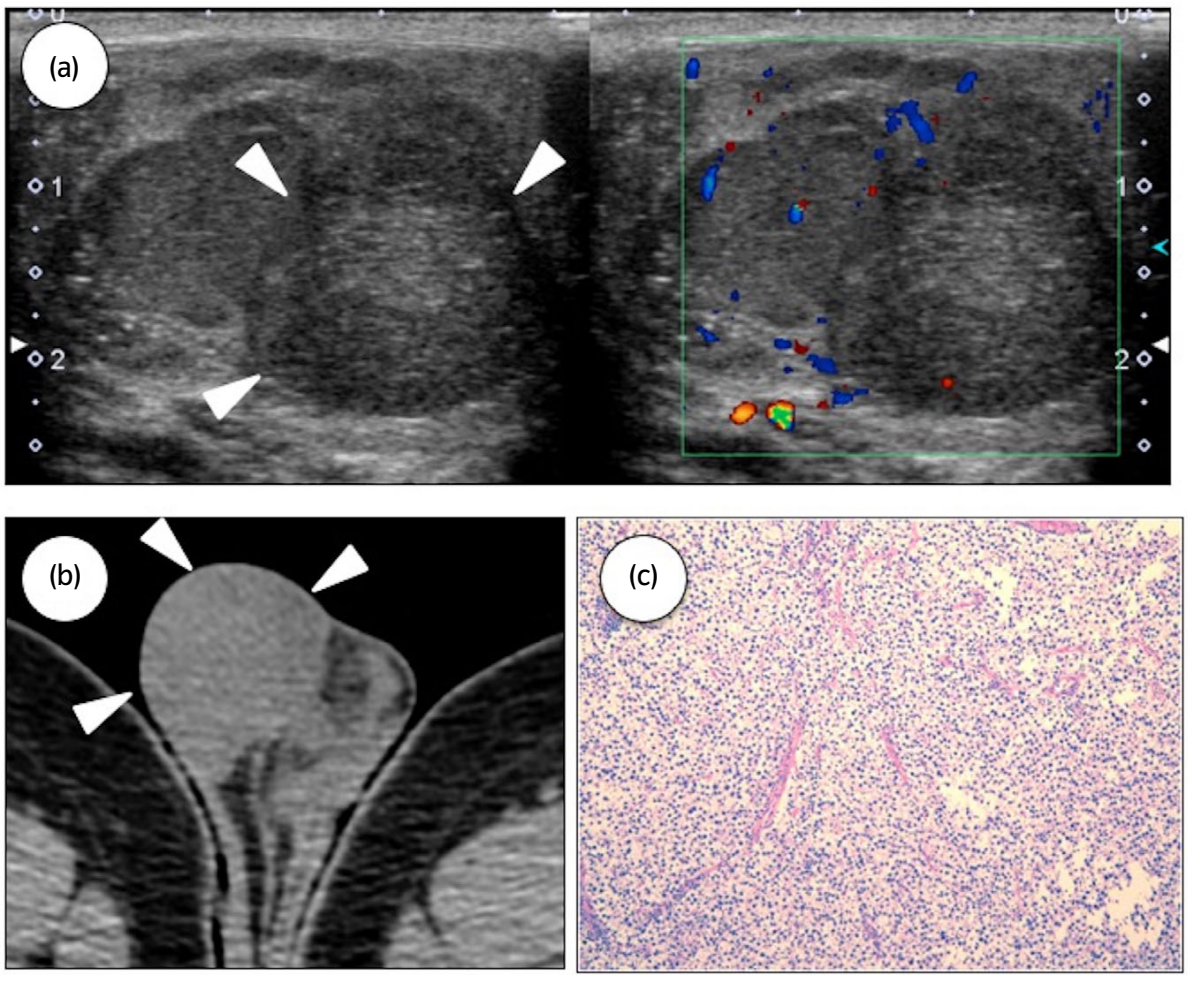
Case 2. (a) Ultrasound examination of the right testicle. A 2 cm tumor inside the testicle is visualized. (b) Computed tomography of the testes. (c) Hematoxylin and eosin staining of the pure seminoma.

There was no history of cryptorchidism in both patients. An elder brother was diagnosed with testicular tumor at the age of 30 years old because of palpable mass in the left testicle (Fig. 1a). There was no family history of testicular cancer or any other malignancy. Among the tumor markers, only AFP was elevated (390 ng/mL). High inguinal orchiectomy was performed. Tumor size was 6 × 5 cm and the pathological diagnosis was stage 1 mixed TGCT (seminoma, yolk sac tumor) (Fig. 1b), no adjuvant treatment was done. No recurrence was observed during the follow‐up period of 4 years and 4 months. A younger brother was cautious about germ cell tumor and he would often perform testicular self‐examination. At the age of 30 years old, 3 years and 6 months since the diagnosis of testicular tumor in elder brother, the younger brother found a lump in the right testicle (Fig. 2a,b). The diagnosis was confirmed by ultrasound examination. Tumor markers were not elevated. High inguinal orchiectomy was performed. Tumor size was 3 × 2 cm and the pathological diagnosis was pure seminoma (Fig. 2c). No recurrence was observed during the follow‐up period of 10 months.

## Discussion

Incidence of the familial testicular tumor ranges from 2% to 5% of all patients with TGCT.^6^ In our institutions it was 3%. Familial testicular tumors occur at earlier age than non‐familial and are often bilateral. More than two members of the same family are affected in rare cases. There are reports on three brothers^7^ and five members of the same family (four brothers and a first cousin on the mother’s side).^7^ There are families with other cancer syndromes which members have also testicular cancer.^7^ The overall risk of the familial testicular tumor is six‐fold, in brothers it is 8‐ to 10‐fold if one brother is affected, sons of men with TGCT have a four‐fold to six‐fold increase in TGCT risk,^8^, ^9^ and risk increases to 40‐fold in identical twins. Reported histology includes embryonal carcinoma, teratoma, choriocarcinoma, and seminoma. In the present cases older brother was diagnoses with mixed TGCT (seminoma, yolk sac tumor) and in the younger one pure seminoma was found.

It has been reported that the FRR for a son whose father has TGCT, is 4.03, and FRR for non‐seminoma is higher than for seminoma.^10^ The lifetime FRR with one affected brother is 5.88. The FRR with two affected brothers is 21.71. The lifetime FRRs of a brother developing TGCT with one affected brother varied from 5.3 to 5.9, depending on birth year, with higher FRR estimates were found for developing TGCT by age 15 years. The lifetime FRRs for a brother having two brothers with TGCT varied from 16 to 22, depending on the number of healthy brothers in the family. Stronger heritable predisposition was observed in non‐seminoma than in seminoma.^10^ The fact that, on average, seminoma is diagnosed at 7‐year‐later age than non‐seminoma, also supports the idea of a stronger heritable component in non‐seminoma TGCT.^11^ Moreover, in other studies, no difference between seminoma and non‐seminoma incidence has been observed.^12^ In our cases, age at diagnosis was the same in both brothers.

Familial TGCT is a multifactorial disease. Genetic predisposition, shared diets, similar environmental exposures, analogous pattern of reproductive decision making might be similar in a certain family. Risk factors for TGCT are heterogeneous including contralateral testicular cancer, cryptorchidism, persistent Müellerian duct syndrome, testicular dysgenesis, infertility, testicular atrophy, testicular intraepithelial neoplasia, mixed gonadal dysgenesis, and Klinefelter's syndrome. Previously published data suppose the presence of a certain pattern of familial TGCT inheritance. Segregation analyses suggest an autosomal recessive mode of inheritance.^13^ Linkage analyses have pointed out on several genomic regions, which could be connected with low‐penetrance TGCT susceptibility. In TGCT the commonest chromosomal abnormality is the formation of isochromosome 12p (extra copy of short arm of chromosome 12). In 2000, the International Testicular Cancer Linkage Consortium reported 8–10 times increased risk of testicular cancer in brothers. A strong linkage was showed with a putative testicular cancer susceptibility locus on chromosome Xq27.^14^ They located a putative TGCT susceptibility gene, that may also predispose to cryptorchidism, to chromosome Xq27. A group from National Cancer Institute modeled the potential discriminatory power of a risk‐SNP‐based algorithm^15^ based on seven SNPs (TERT, CLPTM1L, DMRT1, A7F7IP, SPRY4, BAK1, KITLG) and one microdeletion (chromosome Y AZFc region gr/gr).^13^ Thus, the precise genetic basis for familial TGCT is still to be elucidated. The most possible scenario is that the combined effects of multiple genes with modest risk of testicular cancer may be in the background of familial predisposition for TGCT.

## Conclusion

Testicular self‐examination is recommended for family members of a person with TGCT. A definite genetic abnormality of a familial variant of TGCT has not been identified. Genes are not the only factors of a familial variant of TGCT. Diets, similar environmental and/or occupational exposures, analogous pattern of reproductive decision making might be similar in a certain family. Thus, further research on the genetic predisposition in testicular cancer is necessary.

## Consent

The patients signed written informed consent form for the case to be published.

## Conflict of interest

The authors declare no conflict of interest.

## References

[1] Bosl G , Bajorin D , Sheinfeld J , Motzer R , Chaganti R . DeVita, Hellman, and Rosenberg's Cancer: Principles & Practice of Oncology. Lippincott Williams & Wilkins, Philadelphia, 2008.

[2] Wanderas EH , Fossa SD , Tretli S . Risk of a second germ cell cancer after treatment of a primary germ cell cancer in 2201 Norwegian male patients. Eur. J. Cancer 1997; 33: 244–52. 9135496 10.1016/s0959-8049(96)00459-5

[3] Huyghe E , Matsuda T , Thonneau P . Increasing incidence of testicular cancer worldwide: a review. J. Urol. 2003; 170: 5–11. 12796635 10.1097/01.ju.0000053866.68623.da

[4] Purdue MP , Devesa SS , Sigurdson AJ , McGlynn KA . International patterns and trends in testis cancer incidence. Int. J. Cancer 2005; 115: 822–7. 15704170 10.1002/ijc.20931

[5] Holmes L , Escalante C , Garrison O *et al* Testicular cancer incidence trends in the USA (1975–2004): plateau or shifting racial paradigm? Public Health 2008; 122: 862–72. 18555499 10.1016/j.puhe.2007.10.010 PMC2551560

[6] Heimdal K , Olsson H , Tretli S , Flodgren P , Børresen AL , Fossa SD . Familial testicular cancer in Norway and southern Sweden. Br. J. Cancer 1996; 73: 964–9. 8611416 10.1038/bjc.1996.173 PMC2074255

[7] Ottesen AM , Rajpert‐De Meyts E , Holm M *et al* Cytogenetic and molecular analysis of a family with three brothers afflicted with germ‐cell cancer. Clin. Genet. 2004; 65: 32–9. 15032972 10.1111/j..2004.00180.x

[8] Dong C , Hemminki K . Modification of cancer risks in offspring by sibling and parental cancers from 2,112,616 nuclear families. Int. J. Cancer 2001; 92: 144–50. 11279618

[9] Hemminki K , Li X . Familial risk in testicular cancer as a clue to a heritable and environmental aetiology. Br. J. Cancer 2004; 90: 1765–70. 15208620 10.1038/sj.bjc.6601714 PMC2410275

[10] Valberg M , Grotmol T , Tretli S , Veierød MB , Moger TA , Aalen OO . A hierarchical frailty model for familial testicular germ‐cell tumors. Am. J. Epidemiol. 2014; 179: 499–506. 24219863 10.1093/aje/kwt267

[11] Wanderås EH , Tretli S , Fossrå SD . Trends in incidence of testicular cancer in Norway 1955–2013;1992. Eur. J. Cancer 1995; 31: 2044–8. 10.1016/0959-8049(95)00321-5 8562163

[12] Altieri A , Hemminki K . Number of siblings and the risk of solid tumours: a nation‐wide study. Br. J. Cancer 2007; 96: 1755–9. 17453006 10.1038/sj.bjc.6603760 PMC2359906

[13] Greene MH , Mai PL , Loud JT *et al* Familial testicular germ cell tumors (FTGCT) – overview of a multidisciplinary etiologic study. Andrology 2014; 3: 47–58. 25303766 10.1111/andr.294

[14] Rapley EA , Crockford GP , Teare D *et al* Localization to Xq27 of a susceptibility gene for testicular germ‐cell tumours. Nat. Genet. 2000; 24: 197–200. 10655070 10.1038/72877

[15] Kratz CP , Han SS , Rosenberg PS *et al* Variants in or near KITLG, BAK1, DMRT1, and TERT‐CLPTM1L predispose to familial testicular germ cell tumour. J. Med. Genet. 2011; 48: 473–6. 21617256 10.1136/jmedgenet-2011-100001 PMC3131696

